# Social inequality in morbidity, framed within the current economic crisis in Spain

**DOI:** 10.1186/s12939-015-0217-4

**Published:** 2015-11-14

**Authors:** A.R. Zapata Moya, V. Buffel, C.J. Navarro Yáñez, P. Bracke

**Affiliations:** Centre for Urban Political Sociology and Policies, Universidad Pablo de Olavide, ES-41013 Seville, Spain; Health and Demographic Research (HeDeRa), Department of Sociology, Ghent University, Korte Meer 5, 9000 Ghent, Belgium; Centre for Urban Political Sociology and Policies, Department of Sociology, Pablo de Olavide University, Ctra. de Utrera, km. 1, 41013 Seville, Spain

**Keywords:** Crisis, Fundamental cause theory, Macroeconomic changes, Influence on health, Morbidity, Spain

## Abstract

**Introduction:**

Inspired by the ‘Fundamental Cause Theory (FCT)’ we explore social inequalities in preventable versus relatively less-preventable illnesses in Spain. The focus is on the education-health gradient, as education is one of the most important components of an individual’s socioeconomic status (SES). Framed in the context of the recent economic crisis, we investigate the education gradient in depression, diabetes, and myocardial infarction (relatively highly preventable illnesses) and malignant tumors (less preventable), and whether this educational gradient varies across the regional-economic context and changes therein.

**Methods:**

We use data from three waves of the Spanish National Health Survey (2003–2004, 2006–2007, and 2011–2012), and from the 2009–2010 wave of the European Health Survey in Spain, which results in a repeated cross-sectional design. Logistic multilevel regressions are performed with depression, diabetes, myocardial infarction, and malignant tumors as dependent variables. The multilevel design has three levels (the individual, period-regional, and regional level), which allows us to estimate both longitudinal and cross-sectional macro effects. The regional-economic context and changes therein are assessed using the real GDP growth rate and the low work intensity indicator.

**Results:**

Education gradients in more-preventable illness are observed, while this is far less the case in our less-preventable disease group. Regional economic conditions seem to have a direct impact on depression among Spanish men (y-stand. OR = 1.04 [95 % CI: 1.01–1.07]). Diabetes is associated with cross-regional differences in low work intensity among men (y-stand. OR = 1.02 [95 % CI: 1.00–1.05]) and women (y-stand. OR = 1.04 [95 % CI: 1.01–1.06]). Economic contraction increases the likelihood of having diabetes among men (y-stand. OR = 1.04 [95 % CI: 1.01–1.06]), and smaller decreases in the real GDP growth rate are associated with lower likelihood of myocardial infarction among women (y-stand. OR = 0.83 [95 % CI: 0.69–1.00]). Finally, there are interesting associations between the macroeconomic changes across the crisis period and the likelihood of suffering from myocardial infarction among lower educated groups, and the likelihood of having depression and diabetes among less-educated women.

**Conclusion:**

Our findings partially support the predictions of the FCT for Spain. The crisis effects on health emerge especially in the case of our more-preventable illnesses and among lower educated groups. Health inequalities in Spain could increase rapidly in the coming years due to the differential effects of recession on socioeconomic groups

**Electronic supplementary material:**

The online version of this article (doi:10.1186/s12939-015-0217-4) contains supplementary material, which is available to authorized users.

## Introduction

The severity of the current economic crisis has been much greater in Spain than in other European countries, with the probable exceptions of Portugal, Greece, and Cyprus. This recession has had a significant impact on employment conditions, unemployment levels, deprivation, and poverty rates in the whole of Spain, but with strong differences between the autonomous regions [1]. As a result, Spain at present is a ‘natural laboratory’ for exploring how negative macroeconomic changes influence health and social inequality in health.

Several papers have been published recently, detailing research aimed at investigating the impact of the financial crisis on health and its determinants, especially in those countries hardest hit by the recession [2–7]. Some studies have found influences of the crisis on health in Spain [8–14], while others have found no evidence and claim that health continued to improve during the first years of the crisis [15] or even that recessions are favorable to health [16]. With regard to these apparently contradictory findings, we question whether the impact of macroeconomic conditions–the regional economic context and changes to it–varies across health outcomes and educational levels, the latter being one of the most important SES factors concerning health inequalities.

The recession has had an impact on individuals’ socioeconomic status (SES), through the perception of a reduction in available resources due to job loss, the lowering of wages, and cuts in welfare-state policies and budgets. The Fundamental Cause Theory (FCT) may offer some important guidelines to explore socioeconomic inequalities in differing health outcomes framed within the economic crisis in Spain. This theory states that the association between SES and ill-health is systematically produced as a consequence of the asymmetries in people’s access to a range of ‘flexible resources,’ due to systematic differences in the purposive use of these resources in favor of their own health and because, beyond purposive actions, people can harness indirect health benefits–or not–derived from their socioeconomic position [17, 18].

According to the FCT’s propositions, it is not reasonable to expect that every type of health outcome will be influenced to the same extent by an economic crisis. To analyze whether the crisis influences health, it would therefore be better to focus on different health outcomes for which a degree of preventive knowledge has been developed. It would further be sensible to study these forms of health outcomes in terms of negative impacts as a consequence of the loss of resources. In this way, we can assess conditions in which people can deploy their ‘flexible resources’ to a different extent in times of economic contraction. Moreover, the crisis may have a stronger impact on some socioeconomic groups than on others. For example, the change in unemployment rates from 2007 to 2013, assessed by educational attainment, shows a greater impact on lower-educated groups than on higher-educated ones (Active Population Surveys, National Statistical Institute [APS, 2007–2013]). As a result, the income of the less educated may also be more severely affected. When analyzing the influences of the economic crisis, it is thus necessary to take into account both the differences in the exposure to negative macroeconomic changes and the individual’s capabilities to deal with it.

Inspired by the fundamental cause perspective [19], in this paper we explore regional-level inequalities in highly preventable and relatively less-preventable illnesses. By focusing on education, and framed within the context of the recent economic crisis, we investigate the socio-educational gradient in the occurrence of depression, diabetes, myocardial infarction, and malignant tumors in Spain, and whether this socio-educational gradient varies across the regional-economic context and changes to it.

### Theoretical framework

The FCT is a relevant theoretical contribution from the field of sociology of health and illness. Link and Phelan [19] articulated a theory that tries to explain the persistence of the inverse association between SES and health. The basic notion is that stratification and social inequalities produce an unequal distribution of ‘flexible resources’ (knowledge, money, prestige, power, beneficial social connections, etc.) between individuals and societal contexts, and this ultimately explains the existence and persistence of an inverse association between SES and health outcomes. The theory’s four hypotheses can be summarized as: SES influences multiple illnesses; this influence is through multiple risk factors; SES involves access to flexible resources to avoid or minimize the consequences of illness and; the intervention mechanisms affecting the association between SES and health change over time [20]. Consequently, inequality in health will persist as long as flexible resource inequalities do, and the FCT reveals the inability of interventions focused on eliminating proximal risk factors to eliminate the effects of SES on health. Therefore, flexible resources play a central role in social inequality in health, and operate both at the individual and the contextual level [21].

The main hypothesis of the FCT can be tested by identifying situations where flexible resources cannot help or are less helpful in avoiding or minimizing the consequences of disease. For example, this occurs when information about effective preventive health measures or behaviors is lacking. Accordingly, researchers have tried to test the hypothesis that less-preventable diseases will be associated with SES to a lower extent compared with more-preventable diseases [17, 22–24]. In line with this, our study is based on four health outcomes. First, we selected illnesses that represent important groups of morbidities in terms of the prevalence and cause of mortality. Second, a group of comparatively highly-preventable illnesses (depression, diabetes mellitus, myocardial infarction), and another *relatively* less-preventable morbidity group (in this research, malignant tumors) [23], were chosen. Myocardial infarction is well known as a potentially preventable illness and a large proportion of type 2 diabetes can also be prevented [25]. The field of depression prevention is in the early stages of development, but it is known that there are individual and contextual factors that indirectly help to prevent depression, including cognitive and problem-solving skills, comparatively less-stressful social contexts, working conditions, early-life family conditions, affectivity, and living in an emotionally stable environment [26]. In addition, the prevalence of depression might be moderated through universal interventions or mental health promotion policies. These could improve mental health literacy in the general population and facilitate the recognition of psychological risk and early symptoms. All of the above would contribute to carrying out individual and group targeted interventions to prevent depression [27, 28].

SES is a multidimensional construct comprising diverse factors, including education, employment status, type of work, and economic status [29]. Educational attainment is a notable dimension of SES and it has particular qualities that influence health. It contributes to the improvement of health by means of knowledge accumulated throughout life, enhancing cognitive skills, and amplifying human capital. Ultimately it contributes to increasing an individual’s agency [30]. According to the human capability approach, education not only adds value in production processes, where people can obtain *indirect* benefits (better income, work positions, etc.), but it also has a *direct value* component for people, because it provides capacities to achieve more in leading their life and greater freedom to choose [31]. Associations between education and health have been extensively studied by social-epidemiologists and health sociologists [32, 33]. In addition, the value of education may have risen during recent decades in terms of explaining how health is socially distributed [34]. Two pathways have been identified in the association between education and health: Selection–better health early in life is associated with higher educational attainment–and causation–higher-educated people have better health in adulthood [35]. Further, a range of mechanisms linking education to better health have been identified in relevant literature: good access to healthcare resources, resource substitution or reinforcement advantages, better use of information and innovation, better choices mediated for better life expectancy, healthy preferences such as risk aversion or adopting healthy behaviors, more social support, the positive influence of higher-educated social networks and context, etc. [32, 33, 36].

The various SES factors may have different meanings for different social groups and may affect health outcomes in varying degrees and ways [29]. Accordingly, we explore the influence of a specific component of SES (educational attainment) in line with the following explanatory pathway: Less-educated people are more vulnerable in the current economic context. They have substantial exposure to crisis consequences and in line with the ‘human capital’ and ‘human capability’ approaches, they have fewer possibilities to deal with it. For example, they have reduced opportunities to find a job or improve their SES. This may lead to negative expectations about the near future, and subsequently it can cause feelings of low control over life. By contrast, higher-educated people have accumulated capabilities enabling them to ensure their socioeconomic position is comparatively less affected by the economic crisis. In addition, through education they have acquired the cultural health capital needed to preserve good health, even under stressful conditions. As a result, they are better able to cope with the consequences of the economic crisis, as well as with the health consequences of being vulnerable.

In sum, we test three hypotheses in line with our main objectives. First, we explore the basic prediction which states that SES is a ‘fundamental cause’ of health inequity at individual level. Specifically we test the hypothesis that socio-educational gradients are present in relatively more-preventable illnesses, but not in those that are less preventable where people cannot ‘deploy’ their flexible resources. The second objective is to explore whether macroeconomic context and changes to it have some influence on health outcomes taking into account the previous basic prediction. Regarding to this second objective, our hypothesis states that worse macroeconomic conditions have a negative impact on preventable morbidity, which is an extension of FCT prediction at contextual level. Finally, inspired by a combination of the FCT and the human capability approach, we assess whether macroeconomic changes in a recessionary period have effects on the inverse association between individual SES and health. According to that, our third hypothesis posits that the effects of macroeconomic changes will be stronger in more-preventable illnesses and will be particularly apparent for less-educated people, because in line with the FCT, they will have fewer flexible resources to deal with the negative consequences of the economic crisis and ultimately to protect their health, either through purposive actions or by the harnessing of indirect benefits derived from their SES. Ultimately, we try to assess whether there is evidence that macroeconomic changes during the crisis period have increased social inequality in terms of morbidity, particularly in regions severely hit by the economic crisis.

## Material and methods

### Sample data

We use data from three waves (2003–2004, 2006–2007, and 2011–2012) of the Spanish National Health Survey (SNHS), and the 2009–2010 wave of the European Health Survey in Spain (EHS-S). The SNHS and the EHS-S have a similar cross-sectional design. An extensive methodological description for each survey can be found elsewhere (www.ine.es). These surveys provide representative socio-epidemiological information about the non-institutionalized adult population in 17 Spanish autonomous regions. Respondents were selected using stratified sampling methods across three stages. First, census tract units were selected using weighting depending on demographic strata size. In the second stage, private households were selected using systematic random sampling with an equal probability for each household within each census tract previously selected. Last, one respondent was selected with an equal probability between all the relevant members of the household (≥16 years old in the SNHS and ≥ 15 years old in the EHS-S). Data was gathered via face-to-face interviews.

Our analyses are restricted to respondents aged 25–65, in order to focus on people of working age and to minimize the possibility that they were still in education at the time of the interview. We use two datasets for our analyses: first, a pooled dataset with information from the 2003 and 2011 SNHS and the 2009 EHS-S, to study depression. Second, a dataset with information from the 2006 and 2011 SNHS and 2009 EHS-S, to study diabetes, myocardial infarction and malignant tumors. This decision is due to specific question about the diagnosis of depression was included in the 2006 NHS questionnaire together with occurrences of chronic anxiety; therefore it could not be used as a comparable starting point in the case of depression. The first dataset has a subsample of 20,401 male and 21,954 female respondents, with an accumulated percentage of missing values of 0.36 and 0.28 % respectively. The second dataset has a sample of 21,688 male and 26,768 female respondents, with an accumulated percentage of missing values of 1.87 and 1.01 % respectively. In Additional file 1: Table S1, we provide a description of the sample with the individual variables by period and gender.

### Variables

The four surveys include questions to investigate whether respondents suffered from chronic illnesses or other health problems, and if they had been diagnosed by a doctor. Based on the questions related to *depression*, *diabetes*, *myocardial infarction*, and *malignant tumors*, we construct four dummies as dependent variables (1 = yes; 0 = no).

*Education level* is our key independent variable, which contains five categories based on the highest formal education level achieved (International Standard Classification of Education, 2011 [ISCED]): Illiterate, no diploma, or only primary education (ISCED levels 0 and 1); lower secondary (ISCED level 2); upper secondary (ISCED levels 3 and 4); higher technical education (ISCED level 5); and university studies (ISCED levels 6, 7, and 8 [8 = reference category]).

At the individual level, we control for age, work status, marital status, and household type. *Age group* is derived from a metric variable (age) and classifies respondents into four categories: 25–34 (reference group), 35–44, 45–54, and 55–65. *Period* is a categorical variable recoding the year of interview. It has three categories per dataset: 2003 (reference category), 2009, and 2011 for the first dataset; and 2006 (reference category), 2009, and 2011 for the second. We argue that it is important to take period into account, because by including this variable we can partly control for time trends, such as normal economic cycles or changes to health and social policies. In addition, by using the reference period of 2003 for the first dataset and 2006 for the second, we are able to compare the situation during the economic crisis (the 2009–2011 period), which began in Europe at the end of 2007, with the situation before the recession (2003–2006). *Work status* has four categories: unemployed (reference group), employed, inactive (including students, long-term ill, and retired due to age, health, or other conditions), and homemaker. *Marital status* comprises five categories: married (reference group), single, widowed, separated, and divorced. Finally, *household type* is categorized as one of the following: two adults with children (reference group), one adult living alone, two adults with no children, one adult living with children, more than two adults living with children, and other household types.

*The real Gross Domestic Product* (*GDP*) *growth rate* and *low work intensity indicator* are used as regional-economic context variables, together with changes in these measurements across the periods at regional level, reflecting the strength of macroeconomic changes. These change variables allow exploration of how recession and its negative consequences influence health outcomes within each region. The real GDP rate is an indicator of the economic activity of a region. It reflects the total value of all goods and services produced less the value of goods and services used for intermediate consumption in their production (Eurostat). It is a commonly-used indicator to capture the economic cycle. In addition, the technical definition of a recessionary episode is based on changes in the real GDP growth rate [37]. Low work intensity refers to the percentage of persons who live in households where working-age members had been in paid employment for less than 20 % of the potential working time during the year prior to the interview (http://ec.europa.eu/eurostat/statistics-explained/index.php/Material_deprivation_and_low_work_intensity_statistics#Low_work_intensity). The objective is to capture differences in structural job opportunities between regions during this period. We opted to use *low work intensity* instead of unemployment rate to capture differences at regional labor markets, as the former not only reflects the consequences of the recession on unemployment, but also the intensity of households’ exposure to unstable employment. To construct the context variables, for each region the mean score on the two indicators over the three periods for each dataset is calculated. The change variables are measured for each period within each region and are group-mean centered (abstraction of the group [region] mean), while the above-mentioned regional context effects are grand-mean centered (abstraction of the total mean). In this way, the longitudinal effects of the change indicators at the period level are orthogonal to the cross-sectional effects at the regional level [38, 39]. For both, the context and the change variables, we used external data at regional level (NUTS) of Eurostat, which are shown in Additional file 1: Table S2.

### Statistical analyses

**Fig. 1:**
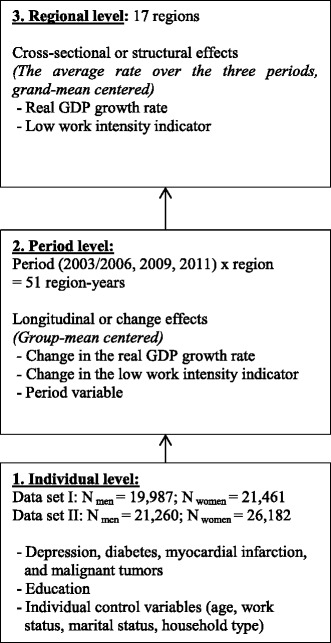
Presentation of the three-level model, with the number of units and the variables per level

We use a micro dataset consisting of a series of repeated cross-sectional sample surveys. Respondents are clustered within periods and regions (Spain has 17 autonomous regions). To obtain an adequate number of higher-level units at the period level–since three periods are not enough to include period as an extra level in our multilevel analyses–we examine the clustering of different waves clustered within regions, as described by Fairbrother [39]. In this way, as presented in Fig. 1, respondents as units on the individual level (Level 1), are nested within region-survey years (Level 2: period level), which are in turn nested within regions (Level 3: region level). In sum, we have a multilevel design of 51 different region-years at the period level, and 17 regions. This multilevel design allows the modelling of cross-sectional effects–or structural effects–to explore between-region differences (at the regional level). In addition, it also allows us to include longitudinal effects–or change effects–in the same model (at the period level), and therefore observe within-region differences along different years [39].

Our analyses consist of two parts: First, we shortly discuss some descriptive results. Table 1 presents the descriptive results for the context and change indicators–the low work intensity indicator and the real GDP growth rate per region. Table 2 shows the percentages of individuals with depression, diabetes, myocardial infarction and malignant tumors, per educational level and period.

**Table 1:** Context and change indicators, real GDP growth rate and low work intensity indicator per region

	Dataset I^a^									
	Real GDP growth rate ($\overline{\mathrm{x}}$)	S.D.	2003-$\overline{\mathrm{x}}$	2008-$\overline{\mathrm{x}}$	2010-$\overline{\mathrm{x}}$	Low work intensity indicator ($\overline{\mathrm{x}}$)	S.D.	2004-$\overline{\mathrm{x}}$	2008-$\overline{\mathrm{x}}$	2011-$\overline{\mathrm{x}}$
Andalucía	1.00	2.71	2.80	−0.20	−2.60	14.50	5.11	−1.20	−4.40	5.60
Aragón	1.27	1.46	1.53	−0.17	−1.37	5.43	1.86	−1.53	−0.53	2.07
Asturias, Principado de	0.93	1.39	1.17	0.37	−1.53	10.70	1.90	−0.50	−1.60	2.10
Balears, Illes	0.40	1.57	0.70	1.10	−1.80	7.43	4.92	−3.23	−2.43	5.67
Canarias	1.43	1.70	1.97	−0.93	−1.03	12.07	6.86	−4.97	−2.87	7.83
Cantabria	0.83	1.17	0.87	0.47	−1.33	8.73	5.12	−5.33	0.47	4.87
Castilla y León	1.23	1.50	1.57	−0.13	−1.43	7.37	1.54	1.03	−1.77	0.73
Castilla-La Mancha	1.07	2.41	2.03	0.63	−2.67	8.27	5.66	−3.27	−3.27	6.53
Cataluña	1.10	1.30	1.50	−0.70	−0.80	7.20	3.82	−2.40	−2.00	4.40
Comunitat Valenciana	0.73	1.62	1.47	0.27	−1.73	8.80	5.05	−3.40	−2.40	5.80
Extremadura	1.57	1.89	1.63	0.43	−2.07	10.50	3.03	−1.80	−1.70	3.50
Galicia	1.40	1.23	0.90	0.50	−1.40	9.70	2.10	0.00	−2.10	2.10
Madrid, Comunidad de	1.20	1.50	1.50	0.00	−1.50	5.10	2.43	−1.20	−1.60	2.80
Murcia, Región de	1.67	2.10	2.03	0.13	−2.17	8.77	4.98	−2.47	−3.27	5.73
Navarra, Comunidad Foral de	1.87	0.97	0.83	0.23	−1.07	3.83	1.02	−0.43	−0.73	1.17
País Vasco	1.63	0.55	0.57	−0.03	−0.53	8.00	1.61	−0.20	−1.50	1.70
Rioja, La	1.70	1.70	1.70	0.00	−1.70	6.97	5.36	−2.37	−3.77	6.13
ᅟ	ᅟ	ᅟ	ᅟ	ᅟ	ᅟ	ᅟ	ᅟ	ᅟ	ᅟ	ᅟ
	Dataset II^b^									
	Real GDP growth rate ($\overline{\mathrm{x}}$)	S.D.	2005-$\overline{\mathrm{x}}$	2008-$\overline{\mathrm{x}}$	2010-$\overline{\mathrm{x}}$	Low work intensity indicator ($\overline{\mathrm{x}}$)	S.D.	2005-$\overline{\mathrm{x}}$	2008-$\overline{\mathrm{x}}$	2011-$\overline{\mathrm{x}}$
Andalucía	0.87	2.50	2.53	−0.07	−2.47	13.63	5.61	−2.93	−3.53	6.47
Aragón	1.40	1.67	1.80	−0.30	−1.50	5.77	1.50	−0.87	−0.87	1.73
Asturias, Principado de	1.20	1.75	1.70	0.10	−1.80	11.13	1.88	0.37	−2.03	1.67
Balears, Illes	1.10	2.33	2.10	0.40	−2.50	6.70	5.74	−4.70	−1.70	6.40
Canarias	1.23	1.36	1.57	−0.73	−0.83	12.40	6.52	−4.30	−3.20	7.50
Cantabria	1.43	2.00	2.07	−0.13	−1.93	9.80	3.54	−3.20	−0.60	3.80
Castilla y León	1.30	1.61	1.70	−0.20	−1.50	7.03	1.29	0.37	−1.43	1.07
Castilla-La Mancha	1.03	2.37	1.97	0.67	−2.63	8.30	5.63	−3.20	−3.30	6.50
Cataluña	1.23	1.53	1.77	−0.83	−0.93	7.17	3.85	−2.47	−1.97	4.43
Comunitat Valenciana	1.07	2.10	2.13	−0.07	−2.07	8.73	5.12	−3.53	−2.33	5.87
Extremadura	1.67	2.02	1.83	0.33	−2.17	11.27	2.61	−0.27	−2.47	2.73
Galicia	1.67	1.56	1.43	0.23	−1.67	9.70	2.10	0.00	−2.10	2.10
Madrid, Comunidad de	1.63	2.18	2.37	−0.43	−1.93	5.10	2.43	−1.20	−1.60	2.80
Murcia, Región de	1.77	2.25	2.23	0.03	−2.27	9.07	4.78	−1.87	−3.57	5.43
Navarra, Comunidad Foral de	1.97	1.11	1.03	0.13	−1.17	4.27	1.02	0.43	−1.17	0.73
País Vasco	2.10	1.32	1.50	−0.50	−1.00	7.83	1.67	−0.53	−1.33	1.87
Rioja, La	1.63	1.60	1.57	0.07	−1.63	6.87	5.43	−2.57	−3.67	6.23

^a^Dataset I: to investigate depression. Real GDP growth rate ($\overline{\mathrm{x}}$= mean of 2003, 2008, and 2010) and low work intensity indicator ($\overline{\mathrm{x}}$= mean of 2004, 2008, and 2011)

^b^Dataset II: to investigate diabetes, myocardial infarction and malignant tumors. Real GDP growth rate ($\overline{\mathrm{x}}$= 2005, 2008, and 2010) and low work intensity indicator ($\overline{\mathrm{x}}$= 2005, 2008, and 2011)

Source: Eurostat and own calculations

**Table 2:** Percent (%) of men and women who suffered from depression, diabetes, myocardial infarction, malignant tumors (diagnosed by a doctor), per region and period

Region	Depression						Diabetes					
	Men			Women			Men			Women		
	2003	2009	2011	2003	2009	2011	2006	2009	2011	2006	2009	2011
Andalucía	3.0	4.3	5.1	7.4	9.8	11.8	6.0	3.9	7.8	5.3	6.3	5.7
Aragón	1.7	2.6	2.6	5.3	5.8	9.0	4.7	6.6	2.6	4.0	4.4	3.7
Asturias, Principado de	8.1	2.5	10.0	14.6	10.7	19.2	5.8	6.5	4.5	4.2	3.9	4.1
Balears, Illes	3.5	3.6	3.9	7.7	11.6	9.9	4.4	2.7	7.3	3.6	3.0	5.0
Canarias	5.6	3.6	7.6	9.0	9.0	15.6	6.6	5.2	8.7	5.7	5.5	5.1
Cantabria	2.8	3.8	4.7	3.7	10.2	7.6	5.0	6.1	6.1	3.4	2.8	2.7
Castilla y León	2.6	5.1	3.5	6.3	7.4	8.9	4.2	4.0	3.8	3.3	2.2	3.7
Castilla-La Mancha	2.7	3.1	3.0	9.1	9.6	16.3	5.5	5.7	6.1	5.0	5.2	5.4
Cataluña	2.3	3.9	4.9	6.9	9.1	9.7	4.4	3.9	4.9	3.5	2.9	4.4
Comunitat Valenciana	2.5	4.5	5.1	6.5	13.4	9.8	5.7	4.9	6.8	4.0	3.8	4.9
Extremadura	1.8	3.9	4.7	14.9	13.7	12.8	7.4	5.1	11.5	5.9	5.9	6.4
Galicia	6.0	6.4	5.9	15.1	17.1	16.1	6.0	5.6	6.8	4.5	4.1	4.5
Madrid, Comunidad de	2.2	3.0	1.9	4.5	7.0	7.0	5.0	5.9	5.1	2.8	2.7	3.4
Murcia, Región de	4.0	7.1	7.3	11.4	16.7	14.2	6.5	6.5	7.3	5.5	6.5	7.3
Navarra, Comunidad Foral de	2.7	2.4	2.0	12.9	4.3	11.8	3.7	5.1	3.3	3.1	3.1	3.3
País Vasco	0.8	2.5	4.4	5.0	4.6	8.1	4.1	4.8	3.4	3.1	3.6	3.5
Rioja, La	2.4	3.0	3.3	5.3	5.8	5.9	5.0	5.1	7.0	2.7	4.0	1.4
ᅟ	ᅟ	ᅟ	ᅟ	ᅟ	ᅟ	ᅟ	ᅟ	ᅟ	ᅟ	ᅟ	ᅟ	ᅟ
Region	Myocardial infarction						Malignant tumors					
	Men			Women			Men			Women		
	2006	2009	2011	2006	2009	2011	2006	2009	2011	2006	2009	2011
Andalucía	3.1	2.1	2.0	0.6	0.6	0.8	1.6	1.6	1.8	2.7	2.7	1.8
Aragón	2.0	3.0	0.8	0.3	0.3	0.4	2.3	2.6	0.8	3.4	2.6	2.4
Asturias, Principado de	3.1	1.8	2.1	1.4	1.4	0.0	1.2	2.2	4.5	2.9	3.9	3.7
Balears, Illes	1.7	2.7	2.6	0.2	1.3	0.8	1.7	1.3	3.4	3.9	4.0	2.3
Canarias	2.0	3.0	2.0	1.3	2.5	0.0	0.7	0.6	1.4	3.1	2.8	5.1
Cantabria	3.0	1.5	1.9	0.9	0.8	1.1	0.6	1.5	2.3	2.3	2.4	4.2
Castilla y León	3.3	2.9	3.5	0.4	0.7	0.3	1.8	1.8	1.5	4.7	3.6	3.1
Castilla-La Mancha	1.8	1.3	1.2	0.5	0.8	0.0	0.7	0.8	1.5	2.5	1.0	3.3
Cataluña	1.5	1.5	1.1	0.6	0.6	0.3	0.9	1.7	2.9	3.9	3.5	2.6
Comunitat Valenciana	1.7	1.1	1.4	0.6	0.2	0.5	1.1	1.7	0.7	1.5	3.1	3.0
Extremadura	1.7	3.3	1.4	1.6	0.3	1.1	1.7	1.5	1.7	3.3	2.2	2.6
Galicia	2.2	2.8	2.0	0.7	0.4	1.0	2.2	1.8	2.8	3.6	4.1	2.3
Madrid, Comunidad de	1.4	1.8	1.4	0.3	0.4	0.1	1.8	2.4	1.1	2.8	2.7	2.6
Murcia, Región de	1.4	1.9	1.5	0.5	0.9	1.4	1.4	3.2	1.9	2.5	5.0	2.8
Navarra, Comunidad Foral de	0.8	1.6	1.2	0.2	0.0	0.0	1.7	0.8	2.5	1.8	1.6	2.6
País Vasco	2.1	2.5	2.4	0.2	0.3	0.8	3.2	3.1	1.9	4.6	2.8	3.8
Rioja, La	1.7	0.5	0.5	0.0	0.4	0.0	1.3	2.0	3.3	2.0	4.9	0.9

Source: Spanish National Health Survey (SNHS) 2003–2004, 2006–2007, and 2011–2012, and European Health Survey in Spain (EHS-S) 2009

Second, to test our hypotheses, logistic three-level analyses are performed, with depression, diabetes, myocardial infarction, and malignant tumors as dichotomous dependent variables. In the first model, we explore and compare socio-educational gradients across models to test the basic prediction of the FCT, while taking the control variables into account (age, marital status, household type, work status, and period). In order to discover how the macroeconomic context and changes therein (the crisis effects) might influence morbidity, we run a second model including the context variables–which aim to reflect the structural economic differences between regions–and the change variables–which try to capture economic change within regions, especially due to the economic recession (Model 2). Last, for each illness where a socio-educational gap is observed, we estimate models including all individual and macroeconomic variables and the cross-level interaction effects of education level with the macroeconomic change variables (Models 3). We have also estimated exploratory models including the cross-level interaction effects between education and macroeconomic context variables, but the most of them are not significant; in addition, these models do not provide essential information in accordance with our third objective. Therefore we have decided to exclude these cross-level interaction terms in order to fit more parsimonious models.

All models are calculated using the MLwiN statistical software package and the Markov Chain Monte Carlo (MCMC) estimation procedure, as this approach has been proven to be suitably robust when also including cross-level interactions [40]. Our analyses are gender stratified and we only consider random intercept models. We use y-standardization, which facilitates the interpretation of results, in particular when Odds Ratios (ORs) are compared across models. In this way, we take unobserved heterogeneity partially into account [41]. We opt for a conservative interpretation of the analyses, due to the large sample size. We ignore all results where *p* > 0.01 for individual effects, and we examine contextual/period and cross-level interaction effects where *p* < 0.1 due to relatively small number of groups at higher levels.

## Results

### Descriptive results

Table 1 presents a synthesis of the macroeconomic context and change variables. We observe that in the period 2003–2010, the mean real GDP growth rate was lower for Illes Balears, Comunitat Valenciana, Cantabria, Principado de Asturias, and Andalucía (≤1 %), and for the period 2005–2010 it was lower for Andalucía, Castilla-La Mancha, Comunitat Valenciana, and Illes Balears (≤1.10 %). It is notable that the change is positive for each region in the first periods (2003 and 2005), while it decreases for some in 2008 and in each region in 2010. This is a clear indication of the strength of the economic crisis in Spain. According to dataset I, Castilla-La Mancha, Andalucía, Region de Murcia, Extremadura, Illes Balears, Comunitat Valenciana, La Rioja, Principado de Asturias, and Comunidad de Madrid are the regions with the strongest decrease in real GDP growth rate (≥1.5 %), compared with the period means for 2003, 2008, and 2010. Dataset II shows a decrease in the real GDP growth rate in 2008 and especially in 2011, compared with the mean GDP growth rate (2005, 2008, and 2010). This decrease is, in particular, observed for the regions Castilla-La Mancha, Illes Balears, Andalucía, Region de Murcia, Extremadura, and Comunitat Valenciana (>2 %). With regard to the low work intensity indicator, the worst structural job opportunities are found in Andalucía, Canarias, Principado de Asturias, and Extremadura (mean of 2004, 2008, and 2011 > 10 %; mean of 2005, 2008, and 2011 > 11 %). The percentage of people living in households with low work intensity increased in 2011 in all regions, but particularly in Canarias, Castilla-La Mancha, La Rioja, Comunitat Valenciana, Region de Murcia, Illes Balears, and Andalucía.

The following table (Table 2) shows that there are cross-regional differences in diagnoses for all the illnesses investigated. The most relevant is that men and women have more diagnoses of depression in 2009 and 2011 than in 2003 in the majority of regions, while the same applies to diabetes in 2011 versus 2006. By contrast, the prevalence of myocardial infarction decreases for men and remains stable for women between 2006 and 2011. With regard to the prevalence of malignant tumors, no clear pattern is found. In some regions, a slight increase from 2006 to 2011 can be observed, while in others there is a decrease.

The last table (Table 3) with descriptive results shows depression, diabetes, myocardial infarction, and malignant tumors by educational level, period, and whether the differences between men and women are significant (using Chi^2^ tests). For each period and most of the educational categories, women have a higher percentage of depression and this gender difference is most pronounced among the lower educational levels. The results reveal an increase in depression from 2003/2006 to 2011, again especially for the less educated. With regard to myocardial infarction, the gender difference is reversed, with men having a higher probability of suffering this illness for each period. In addition, a slight increase from 2006 to 2011 is observed for some educational levels. Furthermore, men with an upper secondary and university degree are more likely to have diabetes than women are, and the prevalence of malignant tumors is higher in some education categories for women in 2006 and 2009. All the above differences in relation to morbidity patterns between men and women justify our decision to perform gender-stratified analyses.

**Table 3:** Gender differences in depression, diabetes, myocardial infarction, and malignant tumors by education level and period

	Depression			Diabetes			Myocardial infarction			Malignant tumors		
2003/2006	M (%)	W (%)	sig. ^a^	M (%)	W (%)	sig.^a^	M (%)	W (%)	sig.^a^	M (%)	W (%)	sig.^a^
Illiterate, no diploma, or primary education	4.2	11.4	***	6.5	7.7		3.3	1.0	***	2.4	3.7	**
Lower Secondary	2.5	7.4	***	2.8	3.6		1.3	0.4	**	1.2	3.4	***
Upper Secondary	2.5	4.7	*	2.4	3.8	*	1.5	0.2	***	1.1	2.6	**
Higher technical education	1.9	4.2		2.1	2.6		1.0	0.1	*	0.6	1.9	*
University Studies	1.5	3.4	**	1.5	2.9	**	1.2	0.3	**	1.2	2.1	*
2009												
Illiterate, no diploma, or primary education	6.8	17.3	***	9.5	7.9		3.6	1.4	***	2.0	4.6	***
Lower Secondary	2.8	9.1	***	2.9	3.3		1.5	0.5	***	1.2	2.5	**
Upper Secondary	2.8	7.2	***	4.7	4.1		1.7	0.5	**	2.1	2.1	
Higher technical education	3.5	4.3		2.3	1.0		0.7	0.4		1.7	1.2	
University Studies	2.3	4.3	**	2.4	1.2	**	1.3	0.1	***	2.1	2.9	
2011												
Illiterate, no diploma, or primary education	8.2	24.9	***	13.2	12.2		3.3	1.4	**	2.3	3.7	
Lower Secondary	5.1	11.9	***	5.9	4.5	*	2.0	0.5	***	2.0	3.0	*
Upper Secondary	4.8	7.5	*	4.8	2.0	***	1.4	0.3	*	2.0	3.4	
Higher technical education	2.3	5.6	**	3.2	3.0		1.1	0.0	*	2.3	2.2	
University Studies	2.0	4.5	***	3.7	1.5	***	0.3	0.1		1.7	1.8	

***p < 0.001 **p < 0.01 *p < 0.5

^a^Difference between men’s and women’s proportion tested via pairwise Chi^2^-test

Source: Spanish National Health Survey (SNHS) 2003–2004, 2006–2007, and 2011–2012, and European Health Survey in Spain (EHS-S) 2009

### Educational gradient in morbidity

First, we test the basic prediction of the FCT: whether the educational gradient is more prominent among highly-preventable illnesses than in those that are less preventable. The logistic regression analyses in Models 1 show socio-educational gradients in depression (Table 4), diabetes (Table 5), and myocardial infarction (Table 6), while as expected by the FCT, not for malignant tumors (Table 7). The likelihood of being diagnosed with depression is higher for less-educated men than for the highest educated (OR = 1.36; OR = 1.28 respectively for the two lowest levels). Women actually have a stronger educational gradient in depression (OR = 1.62; OR = 1.37). The likelihood of being diagnosed with diabetes is also higher for the less-educated (illiterate or only primary education: OR_men_ = 1.41; OR_women_ = 1.67, lower secondary education OR_men_ = 1.21; OR_women_ = 1.35, and upper secondary education OR_men_ = 1.19; OR_women_ = 1.28). With regard to myocardial infarction, an inverse association with educational attainment is observed among men (OR = 1.29; OR = 1.31 respectively for the two lowest levels) and women, but only when the lowest-educated women are compared with the highest (OR = 1.49).

**Table 4:** Depression regressed on education, period, economic context, and change variables, for women and men (y-standardized Odds Ratios)

	Men								Women									
	Model 0		Model 1		Model 2		Model 3b		Model 0		Model 1		Model 2		Model 3a		Model 3b	
	OR		OR		OR		OR		OR		OR		OR		OR		OR	
Intercept	0.18	***	0.07	***	0.08	***	0.07	***	0.31	***	0.10	***	0.11	***	0.10	***	0.10	***
(1) Individual variables^a^																		
Education Level (ref. University Studies)																		
Illiterate, no diploma, or primary education			1.36	***	1.35	***	1.34	***			1.62	***	1.64	***	1.61	***	1.65	***
Lower Secondary			1.28	**	1.27	***	1.23	**			1.37	***	1.38	***	1.37	***	1.40	***
Upper Secondary			1.20	*	1.20	*	1.18	*			1.14	*	1.16	*	1.14	*	1.17	*
Higher technical education			1.16		1.16		1.16				1.08		1.09		1.09		1.11	
(2) Period Variables																		
Period (ref. 2003)																		
2009			1.09		1.07		1.14				1.12	*	1.06		1.08		1.11	
2011			1.13	+	1.11		1.24				1.23	***	1.05		1.13		1.13	
Change in real GDP growth rate					0.99		1.02						0.96		1.05		0.99	
Change in low work intensity indicator					1.00		0.97						1.00		1.01		1.00	
(3) Context Variables																		
Mean real GDP growth rate					0.99		0.96						1.04		1.04		1.05	
Mean low work intensity indicator					1.04	*	1.04	*					1.03		1.04	*	1.03	+
(1) Education level* (2) Period Variables																		
Illiterate, no diploma, or primary education* Change in real GDP growth rate															0.91	**		
Illiterate, no diploma, or primary education* Change in Low work intensity indicator							1.02										1.03	+
Lower Secondary* Change in Low work intensity indicator							1.05	*									1.00	
Variance																		
Region	0.086	(0.056)	0.079	(0.055)	0.055	(0.045)	0.051	(0.043)	0.121	(0.079)	0.084	(0.050)	0.090	(0.059)	0.090	(0.062)	0.084	(0.058)
Period	0.070	(0.038)	0.039	(0.033)	0.044	(0.035)	0.044	(0.041)	0.070	(0.037)	0.046	(0.027)	0.045	(0.024)	0.049	(0.025)	0.050	(0.032)
VPC																		
Region^b^	2.5 %		2.3 %		1.6 %		1.5 %		3.5 %		2.5 %		2.6 %		2.6 %		2.5 %	
Period^c^	2.0 %		1.1 %		1.3 %		1.3 %		2.0 %		1.3 %		1.3 %		1.4 %		1.5 %	
VPC at higher levels (Region + Period)	4.5 %		3.5 %		2.9 %		2.8 %		5.5 %		3.8 %		3.9 %		4.1 %		3.9 %	
DIC:	6467.432		5819.895		5820.183		5819.675		13327.766		12053.531		12055.529		12051.674		12054.975	
Units: Region	17		17		17		17		17		17		17		17		17	
Units: Period	51		51		51		51		51		51		51		51		51	
Units: Individuals	19,986		19,918		19,918		19,918		21,459		21,403		21,403		21,403		21,403	

***p < 0.001; **p < 0.01; *p < 0.05; +*p* < 0.1

N_men_ = 19,987; N_women_ = 21,461

^a^All analyses controlled for age, work status, marital status, and household type at individual level

Model 3a controlled for cross-level interaction effects: change in real GDP growth rate * education level (Not shown); Model 3b controlled for cross-level interaction effect: change in low work intensity indicator * education level (See complete models in Additional file 2)

^b^Variance Partition Coefficient at region level (σ^2^ region)/ (σ^2^ region + σ^2^ period + 3.29)

^c^Variance Partition Coefficient at period level (σ^2^ period)/ (σ^2^ region + σ^2^ period + 3.29)

**Table 5:** Diabetes regressed on education, period, economic context and change variables, for women and men (y-standardized Odds Ratios)

	Men						Women							
	Model 0		Model 1		Model 2		Model 0		Model 1		Model 2		Model 3b	
	OR		OR		OR		OR		OR		OR		OR	
Intercept	0.21	***	0.07	***	0.07	***	0.18	***	0.08	***	0.09	***	0.09	***
(1) Individual variables^a^														
Education Level (ref. University Studies)														
Illiterate, no diploma, or primary education			1.41	***	1.40	***			1.67	***	1.70	***	1.71	***
Lower Secondary			1.21	**	1.20	**			1.35	***	1.37	***	1.38	***
Upper Secondary			1.19	*	1.19	**			1.28	**	1.30	***	1.26	**
Higher technical education			1.04		1.04				1.26	*	1.26	*	1.25	*
(2) Period Variables														
Period (ref. 2006)														
2009			1.01		1.04				1.05		0.94		0.93	
2011			1.13	*	0.96				1.14	**	0.99		1.00	
Change in real GDP growth rate					1.01						0.95		0.94	
Change in low work intensity indicator					1.04	**					0.99		0.97	
(3) Context Variables														
Mean real GDP growth rate					0.96						0.98		0.97	
Mean low work intensity indicator					1.02	*					1.04	**	1.04	**
(1) Education level* (2) Period Variables														
Illiterate, no diploma, or primary education* Change in Low work intensity indicator													1.03	+
Variance														
Region	0.013	(0.013)	0.025	(0.023)	0.019	(0.019)	0.054	(0.030)	0.066	(0.048)	0.026	(0.028)	0.030	(0.028)
Period	0.034	(0.019)	0.016	(0.020)	0.010	(0.012)	0.007	(0.007)	0.005	(0.007)	0.006	(0.007)	0.005	(0.007)
VPC														
Region^b^	0.4 %		0.8 %		0.6 %		1.6 %		2.0 %		0.8 %		0.9 %	
Period^c^	1.0 %		0.5 %		0.3 %		0.2 %		0.1 %		0.2 %		0.2 %	
VPC at higher levels (Region + Period)	1.4 %		1.2 %		0.9 %		1.8 %		2.1 %		1.0 %		1.1 %	
DIC:	8762.19		7419.424		7412.147		8950.835		7963.463		7964.03		7960.65	
Units: Region	17		17		17		17		17		17		17	
Units: Period	51		51		51		51		51		51		51	
Units: Individuals	21,260		21,052		21,052		26,182		25,919		25,919		25,919	

***p < 0.001; **p < 0.01; *p < 0.05; +*p* < 0.1

N_men_ = 21,260; N_women_ = 26,182

^a^All analyses controlled for age, work status, marital status, and household type at individual level

Model 3a controlled for cross-level interaction effects: change in real GDP growth rate* education level (Not shown); Model 3b controlled for cross-level interaction effect: change in low work intensity indicator* education level (Not shown for men). See complete models in Additional file 2

^b^Variance Partition Coefficient at region level (σ^2^ region)/ (σ^2^ region + σ^2^ period + 3.29)

^c^Variance Partition Coefficient at period level (σ^2^ period)/ (σ^2^ region + σ^2^ period + 3.29)

**Table 6:** Myocardial infarction regressed on education, period, economic context, and change variables, for women and men (y-standardized Odds Ratios)

	Men								Women							
	Model 0		Model 1		Model 2		Model 3b		Model 0		Model 1		Model 2		Model 3a	
	OR		OR		OR		OR		OR		OR		OR		OR	
Intercept	0.12	***	0.04	***	0.04	***	0.04	***	0.07	***	0.03	***	0.05	***	0.04	***
(1) Individual variables^a^																
Education Level (ref. University Studies)																
Illiterate, no diploma, or primary education			1.29	**	1.28	**	1.44	**			1.49	**	1.40	*	1.74	**
Lower Secondary			1.31	**	1.31	**	1.43	**			1.27		1.20		1.47	*
Upper Secondary			1.16		1.15		1.28	+			1.10		1.06		1.29	
Higher technical education			1.03		1.03		1.10				0.93		0.89		0.99	
(2) Period Variables																
Period (ref. 2003)																
2009			1.02		1.04		1.11				1.10		0.75		0.69	+
2011			0.88	+	1.01		1.12				0.94		0.63	+	0.55	*
Change in real GDP growth rate					1.02		1.06						0.83	+	1.02	
Change in low work intensity indicator					0.99		0.90	*					0.96		0.96	
(3) Context Variables																
Mean real GDP growth rate					0.99		1.01						1.02		1.05	
Mean low work intensity indicator					1.02		1.02						1.08	**	1.08	***
(1) Education level* (2) Period Variables																
Illiterate, no diploma, or primary education* Change in real GDP growth rate															0.77	+
Lower Secondary* Change in real GDP growth rate															0.75	*
Upper Secondary* Change in real GDP growth rate															0.74	+
Illiterate, no diploma, or primary education* Change in Low work intensity indicator							1.11	*								
Lower Secondary* Change in Low work intensity indicator							1.12	*								
Variance																
Region	0.036	(0.035)	0.016	(0.021)	0.019	(0.024)	0.022	(0.031)	0.096	(0.128)	0.069	(0.088)	0.029	(0.043)	0.021	(0.033)
Period	0.011	(0.015)	0.016	(0.017)	0.006	(0.009)	0.016	(0.023)	0.158	(0.121)	0.072	(0.106)	0.039	(0.039)	0.026	(0.032)
VPC																
Region^b^	1.1 %		0.5 %		0.6 %		0.7 %		2.7 %		2.0 %		0.9 %		0.6 %	
Period^c^	0.3 %		0.5 %		0.2 %		0.5 %		4.5 %		2.1 %		1.2 %		0.8 %	
VPC at higher levels (Region + Period)	1.4 %		1.0 %		0.8 %		1.1 %		7.2 %		4.1 %		2.0 %		1.4 %	
DIC:	4047.762		3448.372		3451.007		3450.421		1851.502		1679.942		1670.216		1673.497	
Units: Region	17		17		17		17		17		17		17		17	
Units: Period	51		51		51		51		51		51		51		51	
Units: Individuals	21,257		21,049		21,049		21,049		26,180		25,917		25,917		25,917	

***p < 0.001; **p < 0.01; *p < 0.05; +*p* < 0.1

N_men_ = 21,260; N_women_ = 26,182

^a^All analyses controlled for age, work status, marital status, and household type at individual level

Model 3a controlled for cross-level interaction effects: change in real GDP growth rate* education level; Model 3b controlled for cross-level interaction effect: change in low work intensity indicator * education level (See complete models in Additional file 2)

^b^Variance Partition Coefficient at region level (σ^2^ region)/ (σ^2^ region + σ^2^ period + 3.29)

^c^Variance Partition Coefficient at period level (σ^2^ period)/ (σ^2^ region + σ^2^ period + 3.29)

**Table 7:** Malignant tumors regressed on education, period, economic context, and change variables, for women and men (y-standardized Odds Ratios)

	Men						Women					
	Model 0		Model 1		Model 2		Model 0		Model 1		Model 2	
	OR		OR		OR		OR		OR		OR	
Intercept	0.12	***	0.08	***	0.08	***	0.15	***	0.07	***	0.08	***
(1) Individual variables^a^												
Education Level (ref. University Studies)												
Illiterate, no diploma, or primary education			0.84	*	0.84	*			0.91		0.92	
Lower Secondary			0.94		0.94				1.02		1.03	
Upper Secondary			0.94		0.94				0.94		0.95	
Higher technical education			0.99		1.00				0.93		0.93	
(2) Period Variables												
Period (ref. 2003)												
2009			1.07		1.05				0.96		0.97	
2011			1.09		0.93				0.87	*	0.85	
Change in real GDP growth rate					0.99						1.00	
Change in low work intensity indicator					1.02						1.01	
(3) Context Variables												
Mean real GDP growth rate					1.13						1.01	
Mean low work intensity indicator					0.99						1.00	
Variance												
Region	0.025	(0.031)	0.018	(0.025)	0.016	(0.024)	0.012	(0.013)	0.019	(0.026)	0.028	(0.036)
Period	0.024	(0.030)	0.029	(0.032)	0.050	(0.041)	0.016	(0.017)	0.017	(0.021)	0.024	(0.022)
VPC												
Region^b^	0.7 %		0.5 %		0.5 %		0.4 %		0.6 %		0.8 %	
Period^c^	0.7 %		0.9 %		1.5 %		0.5 %		0.5 %		0.7 %	
VPC at higher levels (Region + Period)	1.5 %		1.4 %		2.0 %		0.8 %		1.1 %		1.6 %	
DIC:	3795.571		3342.897		3343.505		7058.139		6372.18		6374.839	
Units: Region	17		17		17		17		17		17	
Units: Period	51		51		51		51		51		51	
Units: Individuals	21,257		21,049		21,049		26,181		25,918		25,918	

***p < 0.001; **p < 0.01; *p < 0.05; +*p* < 0.1

N_men_ = 21,260; N_women_ = 26,182

^a^All analyses controlled for age, work status, marital status, and household type at individual level

^b^Variance Partition Coefficient at region level (σ^2^ region)/ (σ^2^ region + σ^2^ period + 3.29)

^c^Variance Partition Coefficient at period level (σ^2^ period)/ (σ^2^ region + σ^2^ period + 3.29)

The cross-level interaction effects between education and change variables (Models 3) were not reported in this table because there is no evidence about educational gradient and contextual/period effects in malignant tumors. In addition these cross-level interaction effects are not significant (See complete models in Additional file 2)

### Regional economic context and change effects on preventable morbidity

If we look at Model 2, where the context and change macroeconomic variables are included, we find that the likelihood of being diagnosed with depression–for men (OR = 1.04)–is stronger in those regions with a higher percentage of people living in households with very low work intensity. In addition, a greater probability of being diagnosed with diabetes is observed for men and women living in regions with low work intensity (OR = 1.02 and OR =1.04 respectively). The same relationship is also found in the case of myocardial infarction for women (OR = 1.08) but not for men. By contrast, there are no significant effects of the macroeconomic context on the likelihood of suffering from malignant tumors.

With regard to the relationship between macroeconomic change and morbidity, no evidence for the whole population (aged 25–65) is found in the cases of depression and malignant tumors. By contrast, there appears to be a positive association between an increase in low work intensity and men’s diagnostics of diabetes (OR = 1.04). In addition, there is a negative association between the real GDP growth rate and myocardial infarction for women: in regions where the real GDP has declined less, women are less likely to suffer myocardial infarction than in regions with a strong decline in the GDP growth rate (OR = 0.83).

### Changes in regional-macroeconomic context and the socioeconomic gradient in preventable morbidity

Next, we extend our exploration to test whether strong negative economic changes–the effects of economic crisis–influence the health of individuals differently depending on their educational level (Hypothesis 3, Models 3).^1^ Our analyses show a negative association between an increase in the real GDP growth rate and the diagnosis of depression for less-educated women (OR = 0.91, Model 3a). This means that in regions with a substantial decline in the GDP growth rate–an indication of a strong crisis effect–the illiterate, women with no diploma, or those with only primary education are more likely to be depressed than those in regions where the GDP growth rate has declined less sharply. In addition, if we look at Model 3b we can see that in regions with higher increase in low work intensity less-educated women and lower secondary men are also more likely to be depressed (OR = 1.03; OR = 1.05, respectively).

With regard to diabetes (Model 3b, Table 5), we see that in regions with an increase in low work intensity, less-educated women are also more likely to have diabetes (OR = 1.03) compared with those in regions with a weaker increase in low work intensity. By contrast, there is no evidence that the negative economic changes influence differently the likelihood to have diabetes according to education level among men.

Furthermore, the educational gradient in myocardial infarction is also associated with macroeconomic change during the recession period. In regions with a strong increase in low work intensity (Model 3b, Table 6), men with a lower or the lowest education level are more likely to suffer from myocardial infarction (respectively OR = 1.12 and OR = 1.11), conversely the increase in low work intensity has apparently a protective effect among those with an university degree (OR = 0.90). This may be an indicator of the rising inequality in myocardial infarction between men during the crisis. In addition, the negative relationship between education and change in the real GDP growth rate for women is also in line with the above finding (Models 3a). Specifically, in regions with a smaller decrease in the real GDP growth rate, women with the three lowest levels of education are less likely to experience a myocardial infarction (OR = 0.77; OR = 0.75; OR = 0.74; respectively) compared with those in regions with a stronger decline in the GDP growth rate.

In addition, some period effects are observed for depression, diabetes, and myocardial infarction. First, baseline Model 1 of Table 4 indicates an increase in women’s depression in 2009 (OR = 1.12) and 2011 (OR = 1.23), compared with 2003. We can also see that men are more likely to suffer from depression in 2011 (OR = 1.13) than in 2003. This increase in the prevalence of depression can mainly be ascribed to the worsening macroeconomic conditions, as these effects are no longer significant after taking context and the macroeconomic change variables into account (Models 2 and 3). Second, in 2011 women and men are more likely to have diabetes than in 2006 (respectively OR _women_ = 1.14; OR _men_ = 1.13; Model 1, Table 5). When we introduce the macroeconomic context and change variables, these period effects are also no longer significant (Model 2). Finally, the probability of being diagnosed with a myocardial infarction decreases for men from 2006 to 2011 (OR = 0.88; Model 1, Table 6).

## Discussion

Before summarizing our main findings, we should address some limitations of this study. First, we use a period design to study crisis effects on chronic morbidity and it is possible that the time periods are too short to capture the full influences of the crisis on illnesses due to their latent stages. Nevertheless, we do find some evidence of an association between economic change and morbidity for specific population groups. Second, due to the cross-sectional design of the study, it is not possible to differentiate between selection and causation pathways. However, this does not detract from our findings, because we know that direct social selection has a minor role in explaining health inequalities and the association between education and health [42]. Further, we are unable to consider income, due to a relatively high percentage of nonresponse, and because the income variable has not yet been verified with other administrative data sources for the 2011–2012 survey. We acknowledge that this is a limitation, as income is a relevant component of SES and can be influenced by the crisis. Nevertheless, the indicators for education and employment situation may at least partially replace any income effects. Last, the use of self-reported data has some well-known limitations [43]. However, self-reported information has been proved robust with regard to studying certain chronic conditions that require continued medical monitoring or ongoing treatment, and this is the case for our health outcomes [44]. In addition, we were unable to compute random coefficient models to see whether educational gradient vary across regions due to limited number of regions at the third level. So we have only considered random intercept models. Regardless of these limitations, our study is the first that uses a multilevel design to investigate the Fundamental Cause Theory within a crisis context and its possible implications for health in Spain. Some very important findings are revealed.

First, our findings partially support the predictions of the FCT in Spain, as we find that education, as a relevant component of SES, has an inverse association with depression, diabetes, and myocardial infarction for both men and women. Conversely, there is no educational gradient concerning the occurrence of malignant tumors, which we use as the *relatively* less-preventable illness outcome. Spain is a very different context to the United States, where FCT emerged with the aim of explaining social conditions as a root cause of the persistence of health inequity, beyond individual risk factors. Recent comparative research has tried to test the theory’s validity for European countries and this has also provided partial support for FCT. It seems that in contexts where there are large inequalities in material resources (such as southern European countries), the contrast between inequality in preventable and non-preventable mortality causes is small or even absent [24]. This is in line with comparative analyses that show relatively less health inequality in southern European countries than other European regions [45–47]. By contrast, we observe moderate educational gradients in some preventable morbidities in Spain, which is not the case for our less-preventable outcome. There are possible explanations for these apparently contradictory findings: First, they could be related to a possible age-cohort effect. During recent decades, Spain experienced a rapid rise in educational attainment due to the implementation of a universal and compulsory education system. Therefore, educational differences between younger and older cohorts are larger at present than in past periods. Accordingly, although we control for age, the emergence and persistence of a gradient in health in Spain could partially reflect this rapid rise in educational attainment between younger cohorts. Second, the emergence of an educational gradient in health, especially in preventable chronic illnesses, could be the consequence of a change in unhealthy behaviors for mainly higher-educated groups. In this regard, some researchers have recently focused on possible explanations for smaller inequities in mortality patterns in Spain [48]. This has been described as a transient situation, attributed to a later socioeconomic modernization process, characterized by little difference in the prevalence of unhealthy behaviors between people with higher and lower SES, and some reverse risk profile in matters such as smoking patterns and alcohol consumption for women during the recent past. This research also shows the existence of a reverse gradient in breast and lung cancer mortality in Spain as a consequence of a previous reverse risk profile [48]. This could partially explain why we do not find an educational gradient with regard to malignant tumors. With our dataset, we are unable to only attribute the non-existence of an educational gradient concerning malignant tumors to their being relatively less preventable, because some preventable types are included in the group. In spite of this, we consider malignant tumors a relatively less-preventable health outcome, because this category does include less-preventable types of cancer. Consequently, more research is needed in order to definitively prove that there is no relationship between SES and other non-preventable illnesses. In line with other researchers [47, 49], we find a greater educational health inequality for women than men in Spain, especially with regard to diabetes and depression. The former can be linked with the finding of Roskam’s study [50], that there is a higher educational gradient in obesity for Spanish women than men. This could ultimately be reflecting the gender-stratified social patterns in diet and physical activity. Our analyses also show higher inequalities in depression for women, which is in line with previous research showing that the largest socioeconomic inequalities in depression are among women in southern European countries [51].

With regard to our second hypothesis concerning the potential influences of the macroeconomic context and changes therein, a direct influence on morbidity is found for depression only among men. The diagnosis of depression is the highest for men in regions with high mean low work intensity, which is in line with previous research showing that mental health problems are higher in countries with a high unemployment rate or unstable work conditions [38, 52]. Our study is the first to show this type of evidence at the regional level, at least in Spain. In addition, in regions with worse structural labor market conditions, women and men are more likely to suffer from diabetes. The same relationship between structural labor market condition and myocardial infarction is found only among women. This could be related to constraints in adopting a healthy diet and other healthy behaviors due to a lack of material and non-material resources. We speculate that there is a possible mechanism linking structural macroeconomic conditions, unpaid household work, household economic resources, leisure time, highly stressful life conditions, and the adoption of unhealthy behaviors. Of course, more research is needed to test whether this mechanism explains social inequality in diabetes and myocardial infarction among women.

With regard to the economic crisis effects on preventable morbidity, we find some interesting associations between the negative changes in macroeconomic conditions and an increasing likelihood of diabetes for men and myocardial infarction for women. These associations are indications of potential influences of the crisis on the increase of cross-regional disparities in two of the most prevalent preventable illnesses in Spain (as expected based on the FCT). Furthermore, this impact is mainly apparent for the less educated (Hypothesis 3): the crisis has a negative impact on less-educated women’s mental health (depression), on lower secondary men’s mental health, and increases the likelihood of myocardial infarction for men and women with the lowest educational attainment. There is no evidence about a direct influence of macroeconomic variables on myocardial infarction for men, by contrast a decrease in the prevalence of myocardial infarction was observed for men between 2006 and 2011 and the associations between macroeconomic variables and myocardial infarction were not significant. In spite of this, our analysis show that educational gap in myocardial infarction among men has expanded in regions where low work intensity increased more during the recession. Simultaneously, in regions with a strong decrease in the GDP growth rate, less-educated women are more likely to suffer from myocardial infarction. We also see that less-educated women are more likely to have diabetes in regions where low work intensity has increased.

These findings show that in some cases a potential effect of the crisis on preventable morbidities could emerge for the whole population, namely, as concerns the association between the increase in low work intensity and diabetes among men. In most cases though, the crisis hits lower socioeconomic groups, as can be observed in the association between macroeconomic changes and the prevalence of diabetes among less-educated women; as well as in the association between a change in the macroeconomic conditions and depression, and myocardial infarction among less educated men and women. Therefore we interpret our findings to support our third hypothesis on the social reproduction of health inequalities through multiple mechanisms, signaling that SES functions as a ‘fundamental cause’.

With regard to our findings concerning myocardial infarction: even when some recent studies have demonstrated a decrease in unhealthy behaviors during economic crisis [53–55], Macy et al. [56] state that this reduction is not equal for all socio-demographic groups. For example, they showed that a change in employment status is associated with a higher likelihood of smoking for people with a level of education below a bachelor’s degree. This suggests that a change in employment status may be more detrimental to the health behavior of the less educated. Accordingly, the increase of the education gradient in myocardial infarction among men could be reflecting a stronger reduction in unhealthy behavior among the well-educated. In addition, the crisis also produces stressful life events, especially for lower socioeconomic groups, due to a sudden loss of resources and an increased job insecurity. These stressful situations combined with relatively fewer capabilities and coping mechanisms, could also explain the increased likelihood of having a myocardial infarction. Some of the factors mentioned above have been previously identified, such as accumulated risk factors and triggers for myocardial infarction [57–61].

Finally, the period effects reflect a worsening of mental health (depression) during the crisis, particularly for lower-educated women. This is in line with previous research showing that patients with anxiety disorders and depression increased in Spain between 2006 and 2010 [62]. Other research has shown that mental health problems have only increased among men during the crisis period [4, 13]. However, studies of this type are restricted to crude period measurements, for example comparing the prevalence of mental ill-health at the start of the economic crisis with its prevalence during the crisis, instead of incorporating actual measurements of economic change due to the crisis while simultaneously controlling for period effects and the average macroeconomic conditions, as we have done in our research.

## Conclusion

In conclusion, evidence is found for an education-health gradient in the Spanish population aged between 25 and 65. However, as expected (Hypothesis 1) based on the Fundamental Cause Theory, educational gradients are only observed for the relatively more-preventable illnesses (depression, diabetes, and myocardial infarction), and not for the less preventable (malignant tumors). We have only found evidence of a direct impact of the crisis on diabetes (men) and myocardial infarction (women), so the results partially support our second hypothesis. By contrast, as claimed in the third hypothesis, the crisis apparently reinforces social inequalities in preventable illnesses, our study confirms that the educational inequalities in the more-preventable morbidities–with the lower educated having a higher chance of becoming ill–vary across the impact of the crisis in the Spanish regions–indicated by a strong increase in the regional low work intensity indicator and a decrease in the real GDP growth rate –. Namely, this negative impact emerges–in particular for myocardial infarction among men and women, and for women’s diabetes or depression–among the lower educational groups. Consequently, we have indications of an increase in socioeconomic (educational) inequality in morbidity, particularly in regions severely hit by the economic crisis. This can lead to important implications for public health policies in Spain. If the crisis is affecting the health of some lower socioeconomic groups and there is no policy strategy to avoid the persistence of this negative effect, health inequalities could increase rapidly in the coming years. More research is needed to explore how austerity policies and budget cuts in the welfare state could influence the situation described above, as this represents the loss of contextual flexible resources and could have a stronger impact on the health of lower socioeconomic groups, because the reduction of these contextual resources could be more detrimental for those groups. This situation also may be contributing to increased socioeconomic inequalities in health.

## Additional files

Additional file 1: Table S1. Description of the sample: the individual variables by period and gender. Table S2. Description of the external data: Real GDP growth and People living in households with very low work intensity by period and regions. (DOCX 33 kb) Additional file 2:
**Extended tables.** (XLSX 8586 kb)

## Abbreviations

FCT: Fundamental Cause Theory
SES: Socioeconomic Status
APS: Active Population Surveys from Spain
EHS-S: European Health Survey in Spain
SNHS: Spanish National Health Survey
ISCED: International Standard Classification of Education
GDP: Gross domestic product
NUTS: Nomenclature of Territorial Units for Statistics
MCMC: Markov Chain Monte Carlo
OR: Odds ratio
SE: Standard error

## Footnotes

1 First, we tested in two separate models the interaction effects with the context variables (education * mean real GDP growth rate and education * low work intensity indicator) and with the change variables (education * change in real GDP growth rate and education * change in low work intensity indicator), and subsequently, both were tested simultaneously in one model. As the effects remain similar, we opted to show them in one model (Model 3), because with four outcome variables we already have a large number of tables and models.
