# Supplementary material for: Social inequality in morbidity, framed within the current economic crisis in Spain
Source: Int J Equity Health. 2015 Nov 14;14:131. doi: 10.1186/s12939-015-0217-4 (PMC4647807; doi:10.1186/s12939-015-0217-4)
Supplement: Additional file 1: Table S1. — Description of the sample: the individual variables by period and gender. Table S2. Description of the external data: Real GDP growth and People living in households with very low work intensity by period and regions. (DOCX 33 kb) [file 12939_2015_217_MOESM1_ESM.docx]

**Table S1. Description of the sample: the individual variables by period and gender.**

|  | **2003** | | **2006** | | **2009** | | **2011** | |
| --- | --- | --- | --- | --- | --- | --- | --- | --- |
|  | **M (%)** | **W (%)** | **M (%)** | **W (%)** | **M (%)** | **W (%)** | **M (%)** | **W (%)** |
| **Depression** | 3.0 | 7.8 | - | - | 3.9 | 9.8 | 4.7 | 11.1 |
| **Diabetes** | - | - | 4.9 | 3.9 | 5.0 | 4.1 | 6.1 | 4.5 |
| **Myocardial Infarction** | - | - | 2.0 | 0.6 | 2.0 | 0.7 | 1.7 | 0.5 |
| **Malignant Tumors** | - | - | 1.6 | 3.1 | 1.8 | 3.1 | 2.0 | 2.8 |
| ***Age Groups*** |  |  |  |  |  |  |  |  |
| ***25-34*** | 23.4 | 23.5 | 22.8 | 20.1 | 21.0 | 20.1 | 19.9 | 20.2 |
| 35-44 | 33.4 | 29.6 | 31.0 | 28.6 | 30.0 | 28.6 | 30.1 | 27.3 |
| 45-54 | 23.1 | 22.3 | 24.3 | 25.5 | 25.6 | 25.5 | 26.1 | 25.9 |
| 55-65 | 20.1 | 24.5 | 21.9 | 25.8 | 23.4 | 25.8 | 23.9 | 26.6 |
| **Education Level** |  |  |  |  |  |  |  |  |
| Illiterate, no diploma, or primary education | 38.5 | 40.7 | 35.4 | 30.2 | 29.5 | 30.2 | 13.4 | 14.2 |
| Lower Secondary | 25.4 | 23.8 | 20.3 | 25.9 | 27.2 | 25.9 | 46.7 | 43.7 |
| Upper Secondary | 11.8 | 10.4 | 15.5 | 13.5 | 15.1 | 13.5 | 13.1 | 13.2 |
| Higher technical education | 7.2 | 5.2 | 8.7 | 6.7 | 8.3 | 6.7 | 8.6 | 7.3 |
| University Studies | 17.0 | 19.9 | 19.7 | 23.7 | 19.8 | 23.7 | 18.2 | 21.6 |
| **Work Status** |  |  |  |  |  |  |  |  |
| Employed | 80.0 | 50.1 | 78.8 | 55.6 | 71.8 | 55.6 | 65.8 | 51.7 |
| Unemployed | 7.0 | 8.8 | 6.8 | 13.8 | 14.6 | 13.8 | 18.4 | 15.8 |
| Inactive population | 12.7 | 7.9 | 13.0 | 9.0 | 12.7 | 9.0 | 15.3 | 13.7 |
| Housewife/husband | 0.4 | 33.1 | 0.2 | 21.1 | 0.3 | 21.1 | 0.4 | 18.7 |
| **Marital Status** |  |  |  |  |  |  |  |  |
| Single | 28.6 | 20.2 | 31.0 | 22.2 | 29.7 | 22.2 | 31.5 | 25.0 |
| Married | 67.0 | 67.3 | 61.4 | 61.7 | 62.5 | 61.7 | 60.1 | 59.3 |
| Widower | 0.8 | 6.5 | 1.4 | 6.1 | 1.2 | 6.1 | 1.3 | 5.3 |
| Separate | 2.5 | 3.7 | 3.3 | 4.1 | 2.6 | 4.1 | 2.6 | 3.7 |
| Divorced | 1.2 | 2.2 | 2.7 | 5.9 | 4.0 | 5.9 | 4.3 | 6.5 |
| **Household type** |  |  |  |  |  |  |  |  |
| One adult alone | 9.3 | 9.2 | 14.6 | 13.9 | 15.9 | 13.9 | 18.8 | 15.8 |
| One adult with child/ren | 0.2 | 2.5 | 0.4 | 7.0 | 1.5 | 7.0 | 1.5 | 8.2 |
| Two adults | 18.8 | 22.1 | 23.0 | 21.6 | 21.8 | 21.6 | 19.7 | 22.4 |
| Two adults with child/ren | 31.1 | 26.1 | 28.4 | 35.9 | 39.3 | 35.9 | 38.4 | 32.7 |
| More than two Adults with child/ren | 11.5 | 11.1 | 2.1 | 2.4 | 1.7 | 2.4 | 2.5 | 4.3 |
| Others | 29.2 | 28.9 | 31.5 | 19.1 | 19.8 | 19.1 | 19.2 | 16.6 |

2003,2009 & 2011 database : N _men_=19987, N _women_=21461(accumulated % of missing values: men=0.36, women=0.28).

2006,2009 & 2011 database : N _men_=21260, N _women_=26182 (accumulated % of missing values: men=1.09, women=1.10).

Source: Spanish National Health Survey (SNHS) 2003-04, 2006-07 & 2011-12 and European Health Survey in Spain (EHS-S) 2009

**Table S2. Description of the external data: Real GDP growth and People living in households with very low work intensity by period and regions.**

|  | Real growth rate of regional GDP at market prices by NUTS 2 regions (% of change on previous year) | | | | People living in households with very low work intensity by NUTS 2 regions ( % of total population aged 0 to 59 years) | | | |
| --- | --- | --- | --- | --- | --- | --- | --- | --- |
|  | 2003 | 2005 | 2008 | 2010 | 2004 | 2005 | 2008 | 2011 |
| Andalucía | 3.8 | 3.4 | 0.8 | -1.6 | 13.3 | 10.7 | 10.1 | 20.1 |
| Aragón | 2.8 | 3.2 | 1.1 | -0.1 | 3.9 | 4.9 | 4.9 | 7.5 |
| Asturias, Principado de | 2.1 | 2.9 | 1.3 | -0.6 | 10.2 | 11.5 | 9.1 | 12.8 |
| Balears, Illes | 1.1 | 3.2 | 1.5 | -1.4 | 4.2 | 2 | 5 | 13.1 |
| Canarias | 3.4 | 2.8 | 0.5 | 0.4 | 7.1 | 8.1 | 9.2 | 19.9 |
| Cantabria | 1.7 | 3.5 | 1.3 | -0.5 | 3.4 | 6.6 | 9.2 | 13.6 |
| Castilla y León | 2.8 | 3 | 1.1 | -0.2 | 8.4 | 7.4 | 5.6 | 8.1 |
| Castilla-La Mancha | 3.1 | 3 | 1.7 | -1.6 | 5 | 5.1 | 5 | 14.8 |
| Cataluña | 2.6 | 3 | 0.4 | 0.3 | 4.8 | 4.7 | 5.2 | 11.6 |
| Comunitat Valenciana | 2.2 | 3.2 | 1 | -1 | 5.4 | 5.2 | 6.4 | 14.6 |
| Extremadura | 3.2 | 3.5 | 2 | -0.5 | 8.7 | 11 | 8.8 | 14 |
| Galicia | 2.3 | 3.1 | 1.9 | 0 | 9.7 | 9.7 | 7.6 | 11.8 |
| Madrid, Comunidad de | 2.7 | 4 | 1.2 | -0.3 | 3.9 | 3.9 | 3.5 | 7.9 |
| Murcia, Región de | 3.7 | 4 | 1.8 | -0.5 | 6.3 | 7.2 | 5.5 | 14.5 |
| Navarra, Comunidad Foral de | 2.7 | 3 | 2.1 | 0.8 | 3.4 | 4.7 | 3.1 | 5 |
| País Vasco | 2.2 | 3.6 | 1.6 | 1.1 | 7.8 | 7.3 | 6.5 | 9.7 |
| Rioja, La | 3.4 | 3.2 | 1.7 | 0 | 4.6 | 4.3 | 3.2 | 13.1 |

Source: Eurostat.

Hyperlink to the table of real growth of regional GDP: <http://ec.europa.eu/eurostat/tgm/table.do?tab=table&init=1&language=en&pcode=tgs00037&plugin=1>.

Hyperlink to table of People living in households with very low work intensity:

<http://appsso.eurostat.ec.europa.eu/nui/show.do?dataset=ilc_lvhl21&lang=en>
